# Long‐term outcome of periprosthetic joint infection following unicompartmental knee arthroplasty: A single‐centre case series

**DOI:** 10.1002/jeo2.70230

**Published:** 2025-04-03

**Authors:** Kevin‐Arno Koch, Johannes Weishorn, Jakob Freytag, Pia‐Elena Frey, Mustafa Hariri, Christian Merle, Tilman Walker

**Affiliations:** ^1^ Department of Orthopaedic Surgery University Hospital of Heidelberg Heidelberg Germany; ^2^ Orthopaedic Centre Paulinenhilfe, Diakonie‐Klinikum Stuttgart Stuttgart Germany

**Keywords:** DAIR, long‐term outcome, periprosthetic joint infection, revision surgery, two‐stage exchange arthroplasty, unicompartmental knee arthroplasty

## Abstract

**Purpose:**

Periprosthetic joint infection (PJI) following unicompartmental knee arthroplasty (UKA) is a rare but serious complication. The data available on this topic are heterogeneous and limited, particularly in regard to long‐term survival and patient‐reported outcomes (PROs). Therefore, the aim of the present study was to analyse the long‐term survival and functional outcome of a case series of PJI following primary UKA at a tertiary referral centre.

**Methods:**

Eighteen knees treated for acute or chronic PJI after primary UKA between 2001 and 2020 with a minimum follow‐up of 2 years were retrospectively identified and evaluated in the present study. Surgical treatment included debridement, antibiotics and implant retention (DAIR) in 10 patients, and two‐stage arthroplasty in 8 patients. Implant survival analysis was conducted using the Kaplan–Meier estimator. Patient‐reported outcome measures (PROMs) were used to assess clinical outcomes.

**Results:**

Overall implant survival free from any revision at 10 years was 83% (95% confidence interval [CI]: 57%–94%). Three DAIR procedures failed due to persistent infection with partially major complications, resulting in a 10‐year revision‐free implant survival of 73% (95% CI: 37%–90%). No reoperation was required in the group that underwent staged treatment. There were no long‐term revisions due to aseptic loosening or degeneration of other compartments in either group. Both groups demonstrated promising median Oxford Knee Scores, with no significant difference (>0.05) between the DAIR (42, range 11–45) and two‐stage exchange arthroplasty (43, range 19–46) groups.

**Conclusions:**

Two‐stage revision procedure offers excellent long‐term survival and high patient satisfaction. The DAIR procedure represents a valid treatment option for acute PJI but carries a certain risk of treatment failure that surgeons should be aware of. Successful treatment of PJI in UKA can provide excellent functional outcomes and long‐term survival without an increased risk of low‐grade infection and aseptic loosening.

**Level of Evidence:**

Level IV.

**Abbreviations**
CI: confidence interval
CR: cruciate retaining
DAIR: debridement, antibiotics and implant retention
EBJIS: European Bone and Joint Infection Society
FU: follow‐up
OKS: Oxford Knee Score
PJI: periprosthetic joint infection
PMMA: polymethylmethacrylate
PRO(M): patient‐reported outcome (measures)
PS: posterior stabilised
ROM: range of motion
SC: semi‐constrained
SD: standard deviation
TKA: total knee arthroplasty
UKA: unicompartmental knee arthroplasty
VAS: visual analogue scale

## INTRODUCTION

Unicompartmental knee arthroplasty (UKA) is an established treatment option for severe isolated unilateral osteoarthritis. According to national registries, the implantation rates for UKA continue to increase [13, 29, 30]. In comparison to total knee arthroplasty (TKA), there is a higher revision rate for UKA, particularly among surgeons with a lower caseload [10, 20, 25]. However, the benefits of UKA are well documented in terms of functional outcome, satisfaction rates and lower perioperative morbidity and mortality [3, 10, 21]. Furthermore, UKA is linked with a reduced likelihood of major complications, including periprosthetic joint infection (PJI) [19, 27, 28]. While PJI occurs in 1%–2% of cases after primary TKA [7, 12, 18], the reported incidence of PJI following UKA is significantly lower, ranging from 0.1% to 0.8% [6, 8, 10, 14].

PJI after primary joint arthroplasty is a rare but devastating complication, that is generally associated with poor functional outcomes and reduced quality of life [4, 15]. The eradication of PJI after UKA is challenging and requires a multidisciplinary approach. The most frequently employed treatment options involve debridement, antibiotics and implant retention (DAIR) for acute PJI and two‐stage exchange arthroplasty for chronic PJI. Despite extensive efforts to prevent PJI over time, a decline in PJI rates could not be observed in recent years [17, 33]. Infection rates after primary UKA are low but have remained stable over the past two decades [8]. Nevertheless, the treatment of PJI is anticipated to increase considerably in line with the rising demand for joint replacement surgery [17]. Increased awareness has led to improved evidence for the treatment of PJI, particularly for TKA. However, data on UKA are scarce and published studies lack long‐term survival and clinical performance [1, 2, 6, 11, 23].

Therefore, the present study aimed to fill this gap by analysing PJI eradication, long‐term survival and patient‐reported outcome (PRO) after treatment of PJI following primary UKA at a tertiary referral centre.

## PATIENTS AND METHODS

### Study cohort

Patients undergoing revision surgery for PJI after medial or lateral UKA at a tertiary referral centre between 2001 and 2020 were screened for eligibility. The study was conducted in accordance with the Declaration of Helsinki of 1975, as revised in 2018, and was approved by the institutional review board prior to patient enrolment. Informed consent was obtained from all participating patients prior to enrolment.

Patients were followed prospectively using the institutional arthroplasty registry and subsequently analysed retrospectively. The diagnosis of PJI was based on a comprehensive evaluation of the patient's medical history, physical examination, laboratory and radiographic findings, and joint fluid aspiration results. Patients were included if they met the European Bone and Joint Infection Society (EBJIS) diagnostic criteria for ‘infection confirmed’ or ‘infection likely’ [24], underwent UKA for primary osteoarthritis, and had a minimum of 2 years of follow‐up. Patients with prior knee infections, severe systemic illness (ASA IV+), or incomplete follow‐up data were excluded. Eighteen patients met the inclusion criteria and were consecutively enrolled in the study cohort. Nine knees underwent the index procedure in‐house, while the remaining nine patients were referred for treatment. Patient demographics and baseline characteristics are shown in Table 1.

**Table 1:** Patient demographics and perioperative patient‐related factors.

Total number of knees	18
Primary diagnosis	
Medial osteoarthritis	16 (88.9%)
Lateral osteoarthritis	2 (11.1%)
Mean age at time of surgery in years (range)	69 (52–54)
Sex	
Men	13 (72.2%)
Women	5 (27.8%)
ASA score	
ASA I	2 (11.1%)
ASA II	7 (38.9%)
ASA III	9 (50.0%)
Charlson Comorbidity Index	
0	8 (44.4%)
1	5 (27.8%)
2	2 (11.1%)
3	1 (5.6%)
4	2 (11.1%)

Abbreviation: ASA, American Society of Anesthesiologists.

A post hoc power analysis was performed to determine the validity of the results. With a large effect size of *ω* = 0.7, an available patient number of *n* = 18, and an *α* of 0.05, the calculated statistical power to detect an underlying difference in clinical outcome was 80%. However, post hoc power analysis indicated that a larger sample size would be required to detect smaller effect sizes with statistical confidence. Given the rarity of PJI following UKA, our study cohort represents a valuable data set.

### Clinical assessment and outcome measures

Clinical and radiographic follow‐up examinations were recommended at regular intervals at 6 weeks, 12 weeks, 1 year, 3 years, 5 years and then every 5 years thereafter. At the most recent follow‐up, the following patient‐related outcome measures (PROMs) were assessed: Oxford Knee Score (OKS), visual analogue scale (VAS) and range of motion (ROM) expressed as the maximum flexion of the knee. Patients who were unable to attend clinical follow‐up were contacted via telephone or mail to complete the questionnaires. In the case of deceased patients, information on further revision procedures or complications between the last clinical follow‐up and death was obtained using information from general practitioners and hospital records.

Treatment success was defined as the absence of further surgical interventions for infection recurrence within 2 years after the initial PJI treatment, as well as the absence of antibiotic suppression therapy [9].

### Surgical treatment and revision protocol

All procedures were carried out at a single institution by several surgeons with special expertise in complex knee arthroplasty. For acute PJI, either post‐operative or haematogenous, and symptom duration ≤4 weeks, DAIR was primarily favoured. For chronic PJI with symptom duration ≥4 weeks and those unresponsive to initial treatment, two‐stage revision procedure was performed. However, treatment allocation was determined on an individual basis in a multidisciplinary setting, in consultation with orthopaedic surgeons, microbiologists and internal medicine specialists.

DAIR procedures involved a complete synovectomy, irrigation with sterile saline and liner exchange. Antibiotic therapy was administered throughout surgical therapy and for 6 weeks after the last operative procedure, according to microbiological susceptibility testing. Two‐stage revision procedures consisted of implant and cement removal, radical debridement of infected and necrotic soft tissue and bone, and insertion of an antibiotic polymethylmethacrylate (PMMA) spacer. Antibiotic therapy was administered for 6 weeks according to sensitivity testing. If persistent infection had been ruled out, TKA was performed. Depending on bone stock, bicondylar or (semi‐)constrained prostheses were used. Antibiotic treatment following reimplantation was administered for a minimum of 6 weeks, in accordance with the results of microbiological resistance testing.

### Statistical analysis

Data were recorded and analysed using Microsoft Excel (Microsoft Corporation), SPSS Version 27.0 (IBM SPSS Statistics), GraphPad Prism Version 10.0 (GraphPad Software) and G‐Power 3.1 (Heinrich Heine University).

The normality of continuous variables was assessed using the Shapiro–Wilk test. As data were non‐normally distributed, non‐parametric Mann–Whitney *U* tests were used for group comparisons. Non‐parametric data were presented as median and range.

Kaplan‐Meier analysis was used to calculate the survival rate of reoperation for ‘any reason’. Censoring was applied for patients lost to follow‐up or deceased due to unrelated causes, ensuring accurate survival estimation. Survival was reported at 2, 5 and 10 years with 95% confidence intervals.

## RESULTS

### Study cohort

All 18 knees (18 patients) that underwent revision for PJI could be followed. The mean follow‐up, excluding revised patients, was 9.2 years (standard deviation [SD] 4.2). Ten knees underwent clinical examination, while three patients were interviewed by phone at the final follow‐up. Two patients died due to unrelated causes and without the need for revision surgery. Three patients underwent revision surgery.

The infection types included 11 cases of acute and 7 cases of chronic PJI. Of the 11 acute PJIs, 8 were attributed to post‐operative infections and 3 to haematogenous infections. Acute post‐operative PJIs occurred after a mean of 17 days (range 4–32) and haematogenous PJIs after 2176 days (range 677–3007) following the index surgery. The mean time between UKA and the initial treatment for chronic PJI was 698 days (range 208–3243).

Of the 18 cases, 13 (72%) tested positive for bacterial growth. Seven patients (39%) who underwent DAIR and six patients (33%) who underwent staged revision had positive cultures. The most frequently isolated bacterial species was *Staphylococcus* (10 out of 13; 77%). Two cases were identified as polymicrobial PJI.

Surgical treatment included DAIR in 10 cases (56%) and two‐stage exchange arthroplasty in 8 cases (44%). One patient (case number 9) with acute PJI was treated with staged revision due to the necessity of infection control in a polytrauma setting and the isolation of a methicillin‐resistant organism. Following the two‐stage revision procedure, four cases underwent secondary TKA, including two with cruciate‐retaining and posterior‐stabilised designs, respectively. In the remaining four two‐stage exchange arthroplasties, semi‐constrained prostheses were utilised. The details of each case are presented in Table 2.

**Table 2:** PJI details and treatment.

Case	Age and gender	Charlson Comorbidity Index	UKA type	Age of implant	Occurrence of symptoms to surgery	Infection type	Infection according to EBJIS	Initial treatment	Organism	Treatment success?
1	65W	1	Medial	5	2	Acute post‐operative	Likely	DAIR	CNS + MSSA	No
2	70M	2	Medial	22	1	Acute post‐operative	Likely	DAIR	Negative	Yes
3	74M	3	Medial	7	4	Acute post‐operative	Likely	DAIR	Negative	Yes
4	69M	0	Medial	21	7	Acute post‐operative	Confirmed	DAIR	MSSE	No
5	69M	0	Lateral	27	1	Acute post‐operative	Confirmed	DAIR	MSSE	Yes
6	52M	0	Medial	4	4	Acute post‐operative	Likely	DAIR	Negative	Yes
7	72M	0	Medial	32	2	Acute post‐operative	Likely	DAIR	*E. coli*	Yes
8	84W	1	Medial	15	1	Acute post‐operative	Confirmed	DAIR	MSSA	No
9	71M	1	Lateral	677	3	Acute hematogenous	Likely	2SE	MRSE	Yes
10	82M	4	Medial	3007	11	Acute hematogenous	Confirmed	DAIR	*E. coli*	Yes
11	61M	1	Medial	2845	4	Acute hematogenous	Confirmed	DAIR	*Streptococcus dysgalactiae*	Yes
12	72W	4	Medial	3243	104	Chronic	Likely	2SE	MSSE	Yes
13	59M	0	Medial	208	157	Chronic	Likely	2SE	MSSA	Yes
14	52M	1	Medial	238	135	Chronic	Likely	2SE	*Staphylococcus haemolyticus*	Yes
15	60M	0	Medial	224	210	Chronic	Confirmed	2SE	MSSA	Yes
16	74W	0	Medial	342	342	Chronic	Likely	2SE	*Acinetobacter lwoffii*/MSSE	Yes
17	79W	0	Medial	215	92	Chronic	Confirmed	2SE	Negative	Yes
18	76M	2	Medial	416	234	Chronic	Confirmed	2SE	Negative	Yes

Abbreviations: 2SE, 2‐stage exchange; DAIR, debridement, antibiotics and implant retention; EBJIS, European Bone and Joint Infection Society; *E. coli*, *Escherichia coli*; CNS, coagulase‐negative Staphylococci; M, man; MRSE, methicillin‐resistant *Staphylococcus epidermidis*; MSSA, methicillin‐sensitive *Staphylococcus aureus*; MSSE, methicillin‐sensitive *Staphylococcus epidermidis*; PJI, periprosthetic joint infection; UKA, unicompartmental knee arthroplasty; W, woman.

### Survival analysis

Three patients (17%) required revision surgery during the study period after failing the DAIR procedure due to persistent infection.

One of these patients underwent an additional DAIR procedure, with the infection subsequently controlled without evidence of persistent PJI at the final follow‐up. Another patient required multiple staged component revisions resulting in an arthrodesis with a stable fistula. The third patient was scheduled to undergo a two‐stage revision arthroplasty. However, following the initial stage of the procedure, which involved the removal of the implant and the implantation of an articulating spacer, the patient experienced a non‐ST elevation myocardial infarction. As a result, the patient was referred to the local cardiology department. Due to the delayed orthopaedic follow‐up, the patient was advised that above‐knee amputation would be the recommended course of action, given the persisting signs of local infection and the compromised local tissue deficiency and health condition. However, the patient and family subsequently declined further operative treatment.

**Figure 1:**
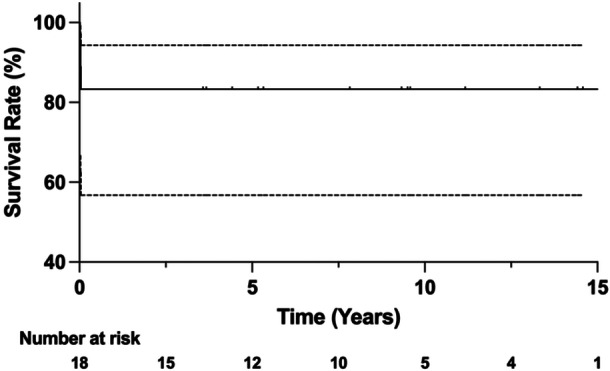
The implant survival rate of revision surgery for any reason estimated with the Kaplan–Meier analysis was 83.3% (95% CI: 56.8%–94.3%) at 2, 5 and 10 years. CI, confidence interval.

The Kaplan–Meier survival analysis with revision for any reason as the endpoint yielded an implant survival rate of 83.3% (95% CI: 56.8%–94.3%) at 2, 5 and 10 years (see Figure 1).

**Figure 2:**
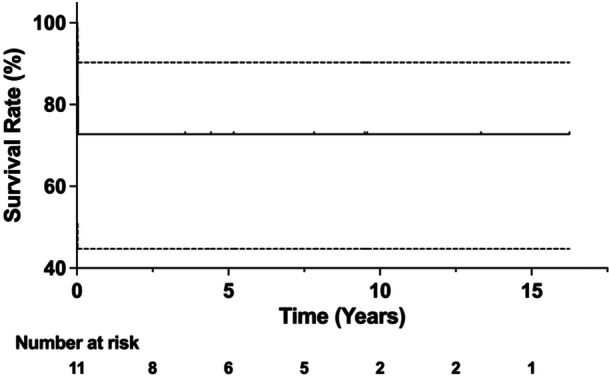
The implant survival rate for patients treated with DAIR for acute PJI estimated with the Kaplan–Meier analysis was 72.7% (95% CI: 37.1%–90.3%) at 2, 5 and 10 years. CI, confidence interval; DAIR, debridement, antibiotics and implant retention; PJI, periprosthetic joint infection.

The 2‐, 5‐ and 10‐year implant survivorship free of any revision for acute post‐operative PJI initially treated with a DAIR procedure was 72.7% (95% CI: 37.1%–90.3%) (see Figure 2). In cases of chronic PJI initially treated with staged component revision, 100% of patients experienced revision‐free survival.

### Clinical outcome

Clinical outcomes were available for 10 patients and assessed for a mean follow‐up of 8.7 years (SD 4.9).

The median OKS was 42 (range 11–45) after DAIR procedure and 43 (range 19–46) after two‐stage exchange arthroplasty, respectively. The OKS differences between DAIR and staged revision were not statistically significant (*p* > 0.05). Of the patients, 70% reported no to low pain levels (VAS < 3), while one patient reported severe pain (VAS 10). Further details on PRO are presented in Table 3.

**Table 3:** PROMs (Oxford Knee Score [OKS], visual analogue scale [VAS] and range of motion [ROM]) at latest follow‐up (mean 8.7 years, SD 4.9).

Case	Age and gender	Treatment	Implant type	OKS	VAS	ROM
2	70M	DAIR	UKA	44	1	n.a.
6	52M	DAIR	UKA	42	2	145
7	72M	DAIR	UKA	45	0	140
10	82M	DAIR	UKA	11	10	70
11	61M	DAIR	UKA	28	3	120
13	59M	2SE	CR	43	0	120
15	60M	2SE	SC	46	0	n.a.
16	74W	2SE	SC	43	0	110
17	79W	2SE	PS	19	5	110
18	76M	2SE	SC	42	0	90

Abbreviations: 2SE, 2‐stage exchange; CR, cruciate‐retaining; DAIR, debridement, antibiotics and implant retention; M, man; n.a., not available; PROM, patient‐reported outcome measure; PS, posterior‐stabilised; SC, semi‐constrained; SD, standard deviation; UKA, unicompartmental knee arthroplasty; W, woman.

## DISCUSSION

The main finding of the present study was that surgical treatment of PJI following UKA results in an overall implant survival of 83% at 10 years. Staged revision surgery with component removal, typically employed to treat chronic PJI, demonstrated a 100% success rate in eradicating infection. The DAIR procedure resulted in 27% of revisions at 10 years, with all cases requiring revision in the early post‐operative period due to persistent PJI. Eradication of PJI resulted in favourable functional outcomes and a low level of pain at mid‐ to long‐term follow‐up.

The occurrence of PJI after primary UKA is a rare but devastating complication. While long‐term clinical outcomes and survival of revision surgery following PJI in TKA are well reported, there is a paucity of clinical studies reporting on the revision rate for PJI in UKA. Furthermore, only two studies have investigated treatment options for infection eradication. The studies in question report an overall infection‐free survivorship following surgical treatment of PJI of 71%–76% at short‐ to mid‐term follow‐ups.

Chalmers et al. report a survivorship free from septic reoperation of 76% at both 1 year and 2 years [6]. With regard to all‐cause reoperation, the survival rate was 71% and 57% at 2 and 5 years, respectively [6]. Hernandez et al. reported a 71% survivorship free of reinfection after PJI treatment following UKA at 5 years [11]. The two‐staged revision procedure demonstrated a 100% success rate at 5 years, whereas the DAIR procedure showed a survival rate of 61% at the same follow‐up point [11]. However, both studies report on small cohorts with short‐ to mid‐term follow‐ups, which may limit the generalisability of the results and underestimate long‐term implant survival. The present study was the first to demonstrate that infection control could be maintained in the long term, with an overall implant survival rate of 83% at 10 years.

In terms of infection eradication, two‐stage exchange arthroplasty is regarded as the gold standard for the treatment of PJI [7, 16]. Previous studies have reported an almost 100% success rate for two‐stage revision surgery for PJI following UKA [5, 6, 11, 26, 31]. The sole instance of PJI recurrence following two‐stage revision was documented by Chalmers et al. without evident rationale, occurring in a patient with no underlying comorbidities and no resistant organisms [6]. Our findings contribute to the existing literature, as the present study did not observe any failure following the two‐stage revision. However, when evaluating DAIR and staged revision procedures, it is essential to consider the significantly higher invasiveness of the procedure with conversion to TKA. This may potentially result in more constrained prostheses and inferior clinical outcomes and patient satisfaction compared to UKA. It is therefore necessary to consider staged revision therapy on an individual basis, with the decision on the most appropriate treatment being made after a detailed analysis of all aspects of PJI management in a multidisciplinary setting.

Although infrequent, reports on DAIR procedures for acute PJI following UKA have become increasingly prevalent in recent years, as evidenced by the literature [1, 2, 6, 11, 23]. The survival rates free from septic revision were found to range between 57% and 84% [1, 2, 6, 11, 23]. However, the available literature presents certain difficulties in terms of comparison, due to the heterogeneous nature of the study cohorts, the diversity of surgical techniques employed, and the varying lengths of the follow‐up periods. For example, a multicentre study by McCormick et al. demonstrated a high infection‐control success rate of 80.8% at 1 year following a DAIR procedure for acute PJI in 52 patients [23]. In contrast, Asadollahi et al. reported a 57% survivorship rate free from septic revision at 5 years for 16 acute PJI cases in a high‐volume, tertiary referral centre [1]. However, the relevance of these studies is limited by the short follow‐up period and limited generalisability.

With a revision‐free survival rate of 73%, the present study is the first and largest to date to provide long‐term data on the success of infection eradication following DAIR in primary UKA. Rapid damage and degradation of native articular cartilage could not be demonstrated in the present study, which challenges previous reports highlighting this entity as a relevant concern after DAIR in PJI following UKA [1, 6, 11, 23]. However, given the size of the cohort in the present study, the relevance of the pathology cannot be dismissed. It is therefore recommended that patients with PJI after UKA should be monitored on a regular basis. All revisions occurred within the early post‐operative period following DAIR, indicating that low‐grade infection and late aseptic loosening may have a limited role in patients undergoing DAIR in the cohort studied. Nevertheless, in the event of unsuccessful DAIR treatment, the persistence of PJI represents a significant and potentially life‐threatening complication in knee arthroplasty surgery [22]. These findings highlight the importance of early intervention in PJI following UKA, which may differ from TKA due to differing implant retention strategies.

The functional outcome after PJI following UKA has not been sufficiently analysed, with only a limited number of studies reporting patient‐related outcome measures. Hernandez et al. provided data regarding the Knee Society Score, indicating excellent post‐operative outcomes with a median score of 94 points at a median follow‐up of 4 years [11]. However, the functional results following the DAIR procedure with UKA and two‐stage exchange arthroplasty with TKA were not differentiated, as they were aggregated. Asadollahi et al. demonstrated excellent functional outcomes following a DAIR procedure for acute PJI, with a median OKS of 45 points at a median follow‐up of 6.5 years [1]. These results were comparable to those observed in uncomplicated primary UKA at the same medical facility [26]. Nevertheless, it is important to exercise caution when interpreting these findings due to the relatively small cohort size, which limits the ability to draw meaningful comparisons. Furthermore, it should be noted that with extended follow‐up periods, there is a possibility of OKS deterioration [32]. In the present study, patients showed excellent functional outcomes at almost 9 years, with a median OKS of 42 after DAIR and 43 after two‐stage exchange arthroplasty. The observed OKS differences between both procedures were not statistically significant. Given an MCID of ~4 points for OKS, this suggests that both treatment groups achieved comparable long‐term functional outcomes.

Although the present results represent the first long‐term evidence on clinical outcomes after PJI in UKA, further research is required in the form of multicentre studies and systematic reviews to provide more meaningful results. This is due to the low incidence of PJI after primary UKA and the resulting small cohort sizes.

The predominant infections were of staphylococcal origin, with *Staphylococcus aureus* and *Staphylococcus* epidermidis representing the most common species. In contrast, gram‐negative bacteria were only sporadically detected. The distribution of isolated organisms in the present study is comparable to that observed in previous studies in the literature [6, 11, 23]. However, the present study revealed a higher proportion of culture‐negative PJI cases. As a tertiary referral centre, this was most likely due to antibiotic use prior to sample taking or loss of information during the process of gathering the microbiological data.

Despite the fact that this is the longest reported follow‐up period regarding the outcome of PJI in UKA and the clinical relevance of the present study, several limitations must be considered when interpreting the data. The retrospective study design is subject to the typical restrictions of this methodology and lacks a comparison group. The diagnosis and treatment options for PJI are subject to constant evolution as new evidence emerges, and the time frame for patient inclusion in a given study may therefore affect the comparability of results. However, the frequency of DAIR and staged revision procedures has remained consistent over time. Furthermore, alterations in the composition of the multidisciplinary team may result in preoperative selection bias. Nevertheless, a multidisciplinary team should be involved in PJI arthroplasty to provide a tailored, individualised treatment plan for the patient, taking into account the best evidence from the different professions to achieve optimal outcomes for this potentially life‐threatening disease. Post hoc power analysis indicated that a larger sample size would be required to detect small and medium effect sizes with statistical confidence. However, given the rarity of PJI following UKA, our study cohort (*n* = 18) represents a valuable data set. It should be noted that the group comparison between DAIR and staged revision may be underpowered for PRO differences due to the relatively small cohort size. However, this is the first study to report long‐term survival and clinical outcomes after PJI at the UKA. Given the rarity of PJI in primary UKA with increasing incidence, a multicentre approach is recommended to provide more robust data. Further studies should also include a separate analysis of DAIR and staged treatment, as published studies lack a differentiated view in this context. Finally, all patients undergoing PJI therapy in the present study were treated in a tertiary referral hospital with special expertise in revision surgery and in a multidisciplinary setting. This may affect the generalisability of the data. Nevertheless, due to the malignant nature of the disease, patients with PJI should be referred to a centre with special expertise in this entity in order to receive the best possible treatment.

## CONCLUSION

PJI represents a rare but devastating diagnosis following primary UKA. The two‐stage revision procedure has been demonstrated to offer excellent long‐term survival, low rates of treatment failure and high patient satisfaction. The DAIR procedure is a valid treatment option for acute PJI, albeit with an inherent risk of treatment failure that surgeons should be aware of. The rate of major complications following DAIR emphasises that the treatment of PJI in primary UKA should be in the hands of experienced centres with a multidisciplinary approach. Successful treatment of PJI in UKA can provide excellent functional outcomes and long‐term survival without an increased risk of low‐grade infection and aseptic loosening.

## AUTHOR CONTRIBUTIONS

**Kevin‐Arno Koch**: Conceptualisation; methodology; investigation; formal analysis; visualisation; writing—original draft preparation; supervision. **Chirstian Merle**: Conceptualisation; methodology. **Tilman Walker**: Conceptualisation; methodology; writing—review and editing; supervision. **Johannes Weishorn**: Investigation; writing – review and editing. **Jakob Freytag**: Investigation; formal analysis; visualisation. **Mustafa Hariri**: Investigation. **Pia‐Elena Frey**: Writing—review and editing.

## CONFLICT OF INTEREST STATEMENT

Christian Merle declares the following conflicts of interest outside the submitted work: Speakers bureau/paid presentations and paid consultant for ZimmerBiomet and Medacta. Financial and material support is provided by LINK, Heraeus and Mölnlycke. Board member/committee appointments for AE Germany. The remaining authors declare no conflicts of interest.

## ETHICS STATEMENT

The current study was approved by the Ethics Commission of the Medical Center, University of Heidelberg (S‐944/2021). Written informed consent was obtained of every patient before inclusion.

## Data Availability

The data will be available upon reasonable request.
